# Risk of retinal vein occlusion in colorectal cancer patients receiving anti-vascular endothelial growth factors – a population-based cohort study

**DOI:** 10.1186/s12885-023-11037-4

**Published:** 2023-06-14

**Authors:** Wan-Ju Annabelle Lee, Wei-Pang Chung, Shih-Chieh Shao, Edward Chia-Cheng Lai, Yi-Chen Chen, Chung-Han Ho

**Affiliations:** 1 Department of Ophthalmology, Chi Mei Medical Center, Tainan, Taiwan; 2 School of Pharmacy, Institute of Clinical Pharmacy and Pharmaceutical Sciences, College of Medicine, National Cheng Kung University, Tainan, Taiwan; 3 Department of Optometry, Chung Hwa University of Medical Technology, Tainan, Taiwan; 4 Department of Oncology, College of Medicine, National Cheng Kung University Hospital, National Cheng Kung University, Tainan, Taiwan; 5 Center of Applied Nanomedicine, National Cheng Kung University, Tainan, Taiwan; 6 Department of Pharmacy, Keelung Chang Gung Memorial Hospital, Keelung, Taiwan; 7 Department of Medical Research, Chi Mei Medical Center, No 901, Zhonghua Road, Yongkang District, Tainan, 710 Taiwan; 8 Department of Information Management, Southern Taiwan University of Science and Technology, Tainan, Taiwan; 9 Cancer Center, Taipei Municipal Wanfang Hospital, Taipei Medical University, Taipei, Taiwan

**Keywords:** Anti-vascular endothelial growth factors, Colorectal cancers, Retinal vein occlusion, Taiwan cancer registry

## Abstract

**Background:**

Anti−vascular endothelial growth factors (VEGFs) treatment has been associated with an increased risk of thromboembolic events. Therefore, the use of anti−VEGFs for patients with colorectal cancers (CRC) has raised concerns about the potential risk of retinal vein occlusion (RVO), an ocular disease caused by embolism or venous stasis. This study aims to evaluate the risk of RVO in patients with CRC treated with anti−VEGFs.

**Method:**

We conducted a retrospective cohort study using the Taiwan Cancer Registry and National Health Insurance Database. The study cohort comprised patients newly diagnosed with CRC between 2011 and 2017, who received anti-VEGF treatment. For each patient in the study cohort, a control group comprising four patients newly diagnosed with CRC, but not receiving anti-VEGF treatment, was randomly selected. A washout period of 12 months was implemented to identify new cases. The index date was defined as the date of the first prescription of anti-VEGF drugs. The study outcome was the incidence of RVO, as identified by ICD-9-CM (362.35 and 362.36) or ICD-10-CM codes (H3481 and H3483). Patients were followed from their index date until the occurrence of RVO, death or the end of the study period. Covariates, including patients' age at index date, sex, calendar year of CRC diagnosis, stage of CRC and comorbidities related to RVO, were included. Multivariable Cox proportional hazards regression models were used to calculate hazard ratios (HRs) with adjustments for all covariates to compare the risk of RVO between the anti-VEGF and control groups.

**Results:**

We recruited 6285 patients in the anti-VEGF group and 37,250 patients in the control group, with mean ages of 59.49 ± 12.11 and 63.88 ± 13.17 years, respectively. The incidence rates were 1.06 per 1000 person-years for the anti-VEGF group, and 0.63 per 1000 person-years for the controls. There was no statistically significant difference in RVO risk between the anti-VEGF and control groups (HR: 2.21, 95% CI: 0.87–5.61).

**Conclusion:**

Our results indicated no association between use of anti-VEGF and occurrence of RVO among CRC patients, although the crude incidence rate of RVO was higher in patients receiving anti-VEGF, compared to control patients. Future study with larger sample size is required to confirm our findings.

**Supplementary Information:**

The online version contains supplementary material available at 10.1186/s12885-023-11037-4.

## Introduction

Colorectal cancer (CRC) is a disease of high prevalence, with an annual incidence over 1.8 million newly diagnosed cases and an annual mortality of almost 700,000 deaths [1]. It is the 3^rd^ most commonly diagnosed cancer worldwide. Although the mortality rate has been decreasing over recent decades, metastatic colorectal cancer (mCRC) is still associated with a poor 5-year survival rate. Before the era of targeted therapies, there were only limited therapeutic options for mCRC. Agents were comprised of a fluoropyrimidine, oxaliplatin or irinotecan, and were used either as single agents or in combinations [2].

Bevacizumab (Avastin®; Genentech, South San Francisco, CA, USA) is a humanized monoclonal antibody that inhibits vascular endothelial growth factor. In randomized, prospective trials, bevacizumab combined with chemotherapy considerably improved response rates, overall survival (OS) and progression-free survival (PFS) in patients with mCRC. Regorafenib (Stivarga®, Beacon Pharmaceuticals Ltd, Bangladesh) is an oral small-molecule multiple kinase inhibitor. In the EU and USA it is indicated for patients with mCRC who have been previously treated with, or who are not considered candidates for available therapies, including fluoropyrimidine-based chemotherapy, anti-VEGF therapy and, if RAS wild-type, anti-epidermal growth factor receptor (EGFR) therapy. Ramucirumab (Cyramza®, Eli Lilly, Toronto, Ontario, Canada) is a fully humanized monoclonal antibody that targets the extracellular domain of the VEGF receptor 2. In the phase III RAISE clinical trial, the addition of ramucirumab to FOLFIRI-based chemotherapy resulted in an improvement of OS in patients with mCRC who had been previously treated with bevacizumab, oxaliplatin and a fluoropyrimidine.

RVO is an ocular emergency situation with sudden visual loss which includes central retinal vein occlusion (CRVO) and branched retinal vein occlusion (BRVO). CRVO occurs when a thrombus occludes the central retinal vein near the lamina cribosa. BRVO occurs when a thrombus develops at the arteriovenous crossing point secondary to atherosclerosis of the retinal artery, causing compression and occlusion of the retinal vein. Given the mechanisms of anti−VEGF agents, retinal vessel occlusion can be seen as a probable adverse event in these patients. Venous thromboembolism is one of the leading causes of morbidity and mortality in patients with malignancy. In previous meta−analysis study, bevacizumab has been reported to be significantly associated with an increased risk of developing venous thromboembolism in cancer patients [3]. Patients treated with bevacizumab had a significantly increased risk of venous thromboembolism with an RR of 1.33 (95% CI: 1.13–1.56; *P* < 0.001), compared with controls. Bevacizumab has been used in the treatment of different cancers for a long time. Retinal vein occlusion (RVO) has been reported as an adverse drug effect of bevacizumab combined with chemotherapy for mCRC [4]. Since there have been very few case reports discussing this association, we aimed to use a large population−based cohort design to determine if anti−VEGF treatment in CRC patients is associated with higher risk of RVO.

## Research design and methods

### Data source

We conducted a new-user design, retrospective cohort study using data from the National Health Insurance Database (NHID), which is a representative population-based sample cohort in Taiwan. The cohort can be used by public health researchers and policymakers to evaluate the effects of medical practice on health outcomes. The NHID covers over 99% of Taiwan’s population and includes information on participants’ insurance eligibility, medical treatment histories, health care provider institutions and general health examinations. Claims are accompanied by diagnostic data collected as ICD-9-CM and ICD-10-CM codes, procedures, prescription drugs, patient personal information, hospital information and locations, direct medical costs of both inpatient and outpatient care and dental services. This study was approved by the Institutional Review Board of Chi Mei Medical Center (11,009-E01). The requirement for written informed consent was waived by the Institutional Review Board of Chi Mei Medical Center due to the de-identified and encrypted nature of personal information in the NHID. Our study was conducted in strict compliance with the ethical standards and guidelines in the 2013 revision of the Declaration of Helsinki. Additionally, to ensure confidentiality and protect the privacy of our participants, our study followed strict guidelines according to the regulations set by the Taiwan Personal Data Protection Act, which was amended on May 26, 2010.

### Study population

**Fig. 1:**
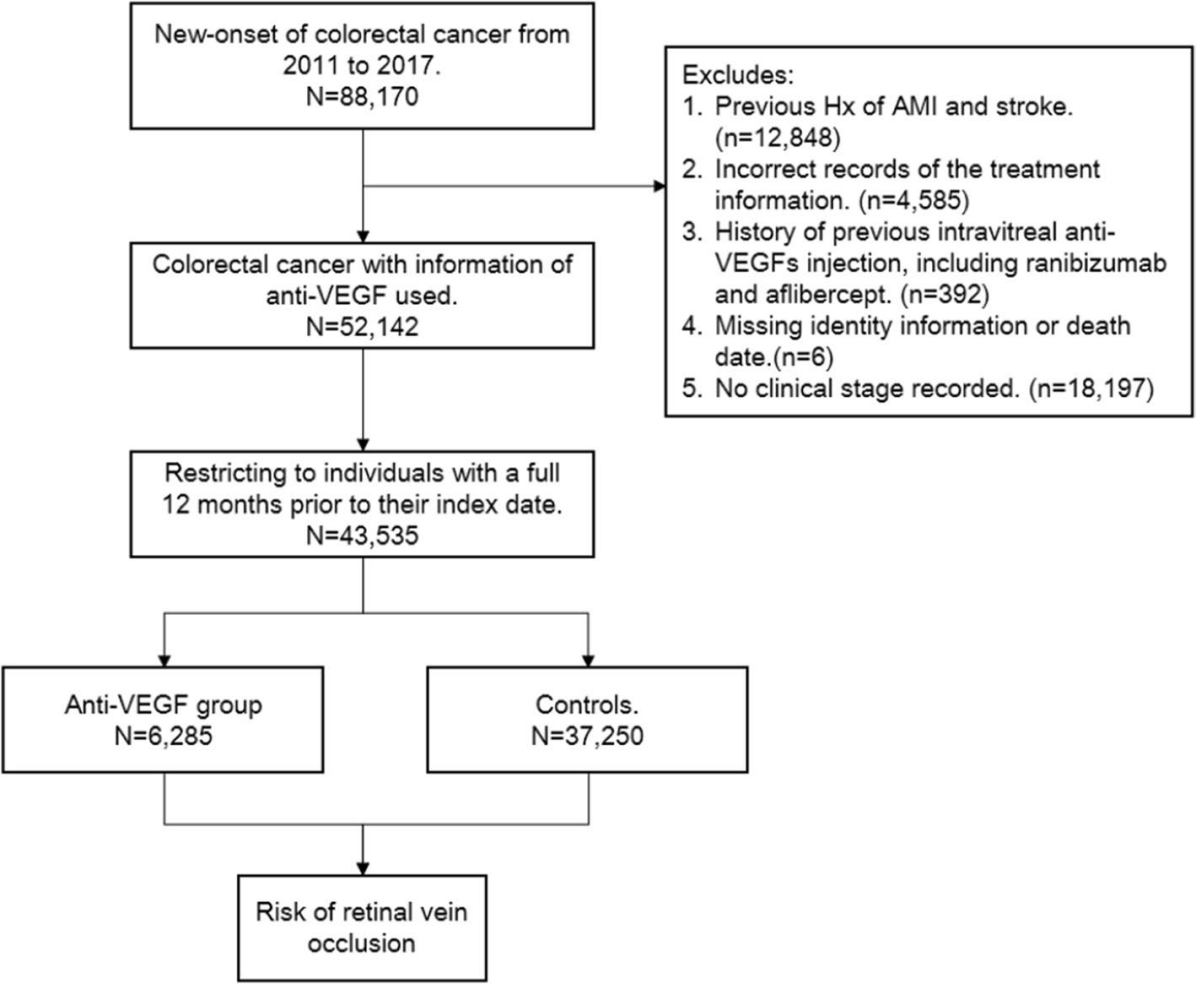
Algorithm of cohort selection

In this study, the Taiwan Cancer Registry (TCR) and Taiwan’s National Health Insurance Database (NHID) were used to identify colorectal cancer diagnosis, associated cancer stage, treatments and baseline comorbidities. Information regarding deaths was recorded in death registration data, which were retrieved from the Ministry of Health and Welfare. For this study, we selected patients with new-onset of colorectal cancer from 2011 to 2017, using ICD-O-3 codes (C18-C20). We defined the index date as the first date of anti-VEGF use. To avoid potential immortal time and selection bias [5], a washout period of 12 months after the cancer diagnosis was imposed in order to assess prevalence of use and to create the anti-VEGF cohort. We therefore included only patients with a full 12 months’ data prior to their index date. The exclusion criteria were as follows: 1) missing date or month of diagnosis; 2) previous retinal vessel occlusion history (including retinal vein occlusion and retinal artery occlusion); 3) previous cardiovascular events, including acute myocardial infarction or stroke, as determined by ICD codes; 4) previous intravitreal use of any anti-VEGF (including ranibizumab or aflibercept); 5) missing data of gender or age; 6) no records of clinical staging of cancer; 7) survival time or follow-up time less than 1 year. For each patient receiving anti-VEGF treatment, four patients not receiving anti-VEGF treatment were randomly selected from the total colorectal cancer patient cohort to form the control group. Figure 1 represents the cohort algorithm.

### Study outcomes and follow-up

Our primary outcome was the incidence of retinal vein occlusion (RVO), as defined by ICD-9-CM (362.35 and 362.36) or ICD-10-CM codes (H3481 and H3483) in the inpatient or outpatient records. Our secondary outcome was all-cause mortality. Patients were followed from their index date until the occurrence of RVO, death or the end of the study period. Patients were censored at the time of the first primary or secondary outcome, on the last date of 2018 or the dropout date from the NHID, whichever came first.

### Definitions of covariates

We retrieved all medical records from the one-year period prior to the date of cancer diagnosis to determine the baseline characteristics of the patients. The covariates were selected according to information from previous studies and experts’ opinions, included patients’ age at index date, sex and comorbidities related to RVO [3, 4, 6, 7]. The comorbidities, including diabetes mellitus (DM), hypertension, dyslipidemia, renal diseases, heart disease, COPD, pneumonia, cataract and glaucoma, were identified using ICD-9-CM or ICD-10-CM codes. All the ICD-9-CM and ICD-10-CM codes are listed in Supplementary Table 1.

### Statistical analyses

Pearson’s chi-squared test was used to compare the demographic characteristics and comorbid disorders between the anti-VEGF and control groups. Variables that could potentially affect treatment assignment or outcomes were selected, including age, gender, diagnosis year, clinical stage and comorbidities (baseline characteristics are listed in Table 1). The disease-free period with regard to the primary outcome and the survival time to all-cause mortality were calculated using Kaplan–Meier curves, while a Log-Rank test was used to analyze intergroup differences. The Cox proportional hazards regression model and incidence rate per 1000 person-years were used to compare the risk of developing an outcome, between the anti-VEGF group and the control group. Hazard ratios (HRs) with 95% CIs were estimated for the comparative risk of outcome between the two groups. Besides adjusting for the covariates of age, gender, clinical stage and history of comorbidities, we made a further adjustment using mortality as a competing event to evaluate the sub-distribution hazard ratio of retinal vein occlusion based on Fine and Gray’s competing risk approach, because the mortality varied with cancer stage and therapy used. Sensitivity analysis also demonstrated the risk of new-onset retinal vein occlusion in colorectal patients without a history of retinal vein occlusion in the two groups. Statistical significance was based on an alpha level of 0.05 in the two‐tailed test. All analyses were performed using SAS V.9.4 (SAS Institute, Cary, NC, USA). Kaplan–Meier curves were generated using Stata version 12 (Stata Corp, College Station, TX, USA).

**Table 1:** Demographic information of colorectal cancer patients receiving anti-VEGF treatment and controls. (*N* = 43,535)

	Anti-VEGF group (*n* = 6285)	Controls (*n* = 37,250)	*P*-value
**Age group, year n (%)**			
< 50	1463 (23.28)	5878 (15.78)	< .0001
51–60	1835 (29.20)	9167 (24.61)	
61–70	1754 (27.91)	9940 (26.68)	
71–80	1010 (16.07)	8029 (21.55)	
81 +	223 (3.55)	4236 (11.37)	
Age, mean ± SD	59.49 ± 12.11	63.88 ± 13.17	< .0001
**Sex, n (%)**			
Male	3526 (56.10)	20,880 (56.05)	0.9433
Female	2759 (43.90)	16,370 (43.95)	
**Diagnosis year, n (%)**			
2011	801 (12.74)	4920 (13.21)	0.0520
2012	935 (14.88)	5176 (13.90)	
2013	869 (13.83)	5113 (13.73)	
2014	848 (13.49)	5266 (14.14)	
2015	833 (13.25)	5193 (13.94)	
2016	982 (15.62)	5427 (14.57)	
2017	1017 (16.18)	6155 (16.52)	
**Initial clinical stage, n(%)**			< .0001
0–1	133 (2.12)	11,101 (29.80)	
2	299 (4.76)	7729 (20.75)	
3	1062 (16.90)	15,883 (42.64)	
4	4791 (76.23)	2537 (6.81)	
**RVO, n (%)**	9 (0.14)	69 (0.19)	0.4660
**History of RVO, n (%)**	15 (0.24)	102 (0.27)	0.6184
**Comorbidity, n (%)**			
Diabetes mellitus	1074 (17.09)	7481 (20.08)	< .0001
Hypertension	1897 (30.18)	13,750 (36.91)	< .0001
Cataract	289 (4.60)	2343 (6.29)	< .0001
Glaucoma	106 (1.69)	811 (2.18)	0.0122
Renal diseases	242 (3.85)	2501 (6.71)	< .0001
Dyslipidemia	941 (14.97)	6942 (18.64)	< .0001
COPD	87 (1.38)	757 (2.03)	0.0006
Heart failure	102 (1.62)	1136 (3.05)	< .0001
Asthma	132 (2.10)	966 (2.59)	0.0211
Liver diseases	314 (5.00)	2051 (5.51)	0.0989
Pneumonia	94 (1.50)	903 (2.42)	< .0001
Arrhythmia	158 (2.51)	1490 (4.00)	< .0001

*P*-value was calculated from Pearson’s chi-squared test for categorical variables

*VEGF* vascular endothelial growth factor, *RVO* retinal vein occlusion

### Sensitivity analysis

To address the imbalance in clinical stages and mortality between the anti-VEGF and control groups, we performed subgroup analyses. First, we used the patients aged under 50 years old as the reference group to compare the HR between different age groups. Second, we used clinical stages 0–1 as the reference group to compare the HR between different stages. Third, besides adjusting for the covariates of age, gender, clinical stage and history of comorbidities, we made an adjustment using mortality as a competing risk to recheck the HR, because the mortality rate between these two groups was different. Fourth, we performed an additional subgroup analysis in which we retrieved those with a history of RVO as a subgroup to calculate the hazard ratios because those patients might have a higher risk of recurrent RVO after anti-VEGF treatment. Finally, we excluded those who had RVO history and checked the new-onset RVO hazards to compare the results. We also performed cause-specific hazard models to determine the competing risk by mortality. Statistical significance was based on an alpha level of 0.05 in the two‐tailed test. All analyses were performed using SAS V.9.4 (SAS Institute, Cary, NC, USA).

## Results

### Demographic data

We selected patients with new-onset colorectal cancers from 2011 to 2017 according to our methods (N = 91,222). After excluding those with missing diagnosis date or month information we had 88,170 cases. We also excluded patients with the following conditions: (1) previous history of acute myocardial infarction and stroke (N = 12,848); (2) lack of correct timing of treatment (N = 4585); (3) history of previous intravitreal injections of anti-VEGFs (including ranibizumab and aflibercept) (N = 392); (4) missing ID information or death date (N = 6); and (5) no clinical staging (N = 18,197). We thus had 52,142 patients, diagnosed with colorectal cancers and with definite initial clinical staging data. We restricted patients to those surviving more than one year after colorectal cancer diagnosis and excluded those who were followed up for less than one year (N = 8607). Finally 43,535 patients with colorectal cancer were enrolled in the study cohort. We then divided these patients into 2 groups for comparison; the anti-VEGF group, receiving anti-VEGF treatment (N = 6285), and the control group, not receiving anti-VEGF treatment (N = 37,250). The index date was defined as the first date of any anti-VEGF use. A flowchart is shown in Fig. 1.

Table 1 shows the demographic characteristics and comorbid disorders of the anti-VEGF group and the controls. The mean ages were 59.49 ± 12.11 years and 63.88 ± 13.17 years for the anti-VEGF and control groups, respectively. Of the 6285 patients in the anti-VEGF group, 3526 (56.10%) were men and 2759 (43.90%) were women, with 1463 (23.28%) under 50 years old, 1835 (29.20%) aged 51– 60 years, 1754 (27.91%) aged 61–70 years, 1010 (16.07%) aged 71–80 years and 223 (3.55%) aged ≧ 81 years old. The year of diagnosis was very similar for the anti-VEGF and control groups (*p* = 0.052). However, the anti-VEGF group had more advanced initial clinical stages; i.e., more than 70% of the patients in this group were at stage 4, while most of the patients in the control group were below stage 3. According to Taiwan’s National Health Insurance regulations, only patients with metastatic colon cancer are able to access anti-VEGFs for their disease treatment. Therefore, we assumed all patients in the anti-VEGF group to have stage 4 disease, since they were receiving anti-VEGFs. Furthermore, the mortality rate in the anti-VEGF group was 68.81%, but only 15.79% in the control group. As for comorbidities, the control group had a higher prevalence of previous comorbidities, such as hypertension, pneumonia, diabetes, dyslipidemia, arrhythmia, heart failure, renal diseases and cataract.

### Incidence rates for RVO

**Fig. 2:**
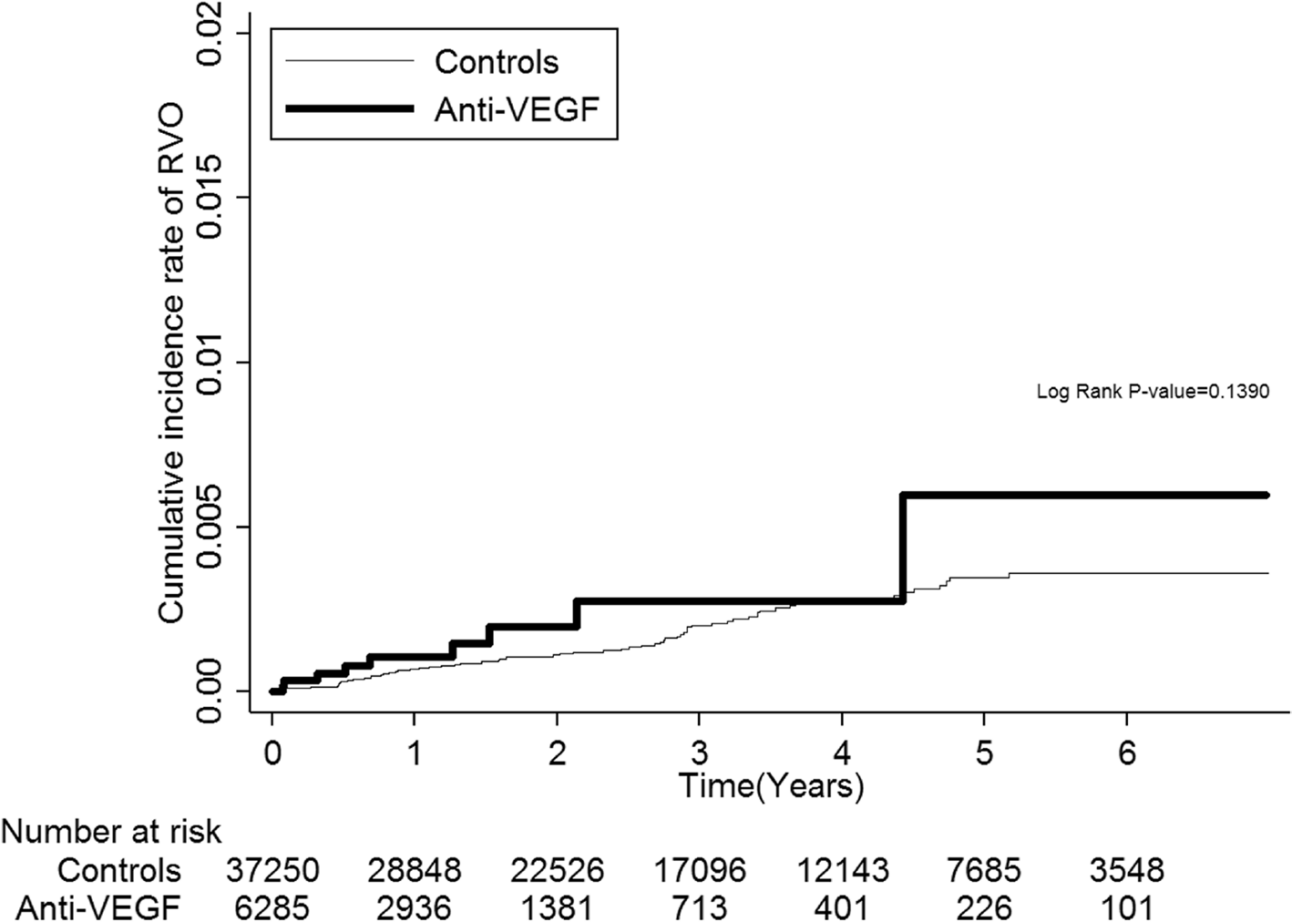
Cumulative incidence rate of retinal vein occlusion

**Fig. 3:**
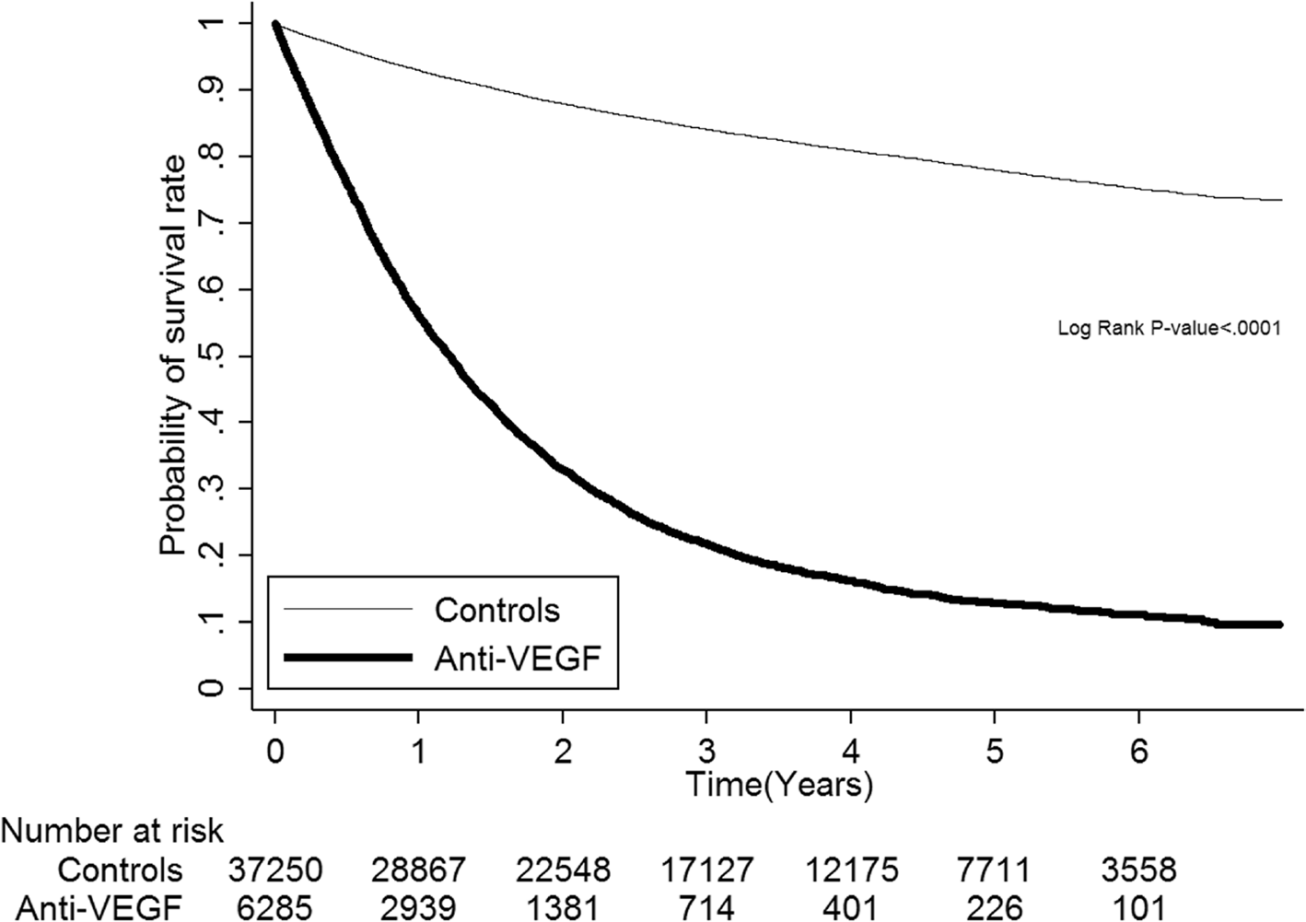
Probability of survival rate

During the follow-up period, 78 patients developed RVO. No significant difference was observed between the two groups, based on proportionality (anti-VEGF group: 9 (0.14%); controls: 69 (0.19%), Table 1). However, the incidence rate was higher in the anti-VEGF group (1.06 per 1000 person-years) than in the control group (0.63 per 1000 person-years), though this result did not reach statistical significance (adjusted HR: 2.21, 95% CI: 0.87–5.61). For all-cause mortality, the anti-VEGF group had a much higher incidence (507.73 per 1000 person-years) than the control group (53.58 per 1000 person-years) (Table 2). Kaplan–Meier analyses demonstrated no higher cumulative incidence rates of RVO in the anti-VEGF group than in the control group (Log Rank *p* = 0.1390 in Fig. 2). Figure 3 shows the probability of survival rate, revealing lower survival in the anti-VEGF group, compared to the control group (Log Rank *p* < 0.001).

**Table 2:** Evaluation of the risk of retinal occlusion

	Anti-VEGF group		Controls		Hazard ratio (95% CI)	
	Number of cases	Incidence rate^a^	Number of cases	Incidence rate^a^	Crude	Adjusted**
Total retinal vein occlusion	9	1.06	69	0.63	1.69 (0.84–3.43)	2.21 (0.87–5.61)
All-cause mortality	4325	507.73	5883	53.58	8.31 (7.98–8.65)	4.69 (4.41–4.98)

^a^Per 1000 person-years

^**^Adjusted for covariates of age, gender, clinical stage and history of comorbidities

*VEGF* vascular endothelial growth factor

### Sensitivity analysis

Significant risk factors for RVO included age 71–80 years (adjusted HR: 3.72, 95% CI: 1.24–11.12), age ≧ 81 years (adjusted HR: 4.74, 95% CI: 1.45–15.47) and history of RVO (adjusted HR: 61.45, 95% CI: 30.93–122.11), whereas gender, clinical stage and all comorbidities were not independent risk factors for RVO. For the competing risk of mortality, we conducted a subgroup analysis using mortality as a competing event, whereby, with the exception of cataract history (adjusted HR: 1.92, 95% CI: 1.09–3.04), the results remained robust and compatible with the primary results. Table 3 presents the crude and adjusted hazard ratios and 95% CIs for RVO during the follow-up period for the study cohort. We used the cause-specific hazard model to compare the risks between the anti-VEGF and control groups, and the adjusted hazard ratio (AHR) was 2.21 (95% CI: 0.87–5.61) for RVO and 4.70 (95% CI: 4.42–4.99) for death. We also checked for new onset RVO by excluding those who had RVO histories, and the result remained consistent with our primary result (adjusted HR: 1.12, 95% CI: 0.45–2.79). In the cause-specific hazard model, the AHRs for new-onset RVO and death were 2.04 (95% CI: 0.75–5.52) and 4.68 (95% CI: 4.41–4.97), respectively (Table 4).

**Table 3:** Adjusted hazard ratios (AHR) and 95% confidence interval (95% CI) for retinal vein occlusion during the follow-up period for the study cohort

	AHR (95% CI)	*p*-value
**Cause-specific hazard regression for RVO**		
**Anti-VEGF group vs. control group**	2.21 (0.87–5.61)	0.0939
**Age group**		
< 50	Ref	
51–60	2.05(0.67–6.26)	0.2080
61–70	2.92(0.99–8.62)	0.0525
71–80	3.72(1.24–11.12)	0.0187
81 +	4.74(1.45–15.47)	0.0099
**Sex, n (%)**		
Male	1.17(0.74–1.85)	0.5055
Female	Ref	
**Initial clinical stage**		
0–1	Ref	
2	1.03(0.54–1.96)	0.9270
3	1.02(0.58–1.77)	0.9554
4	0.97(0.36–2.64)	0.9562
**Comorbidity**		
Diabetes mellitus	1.21(0.68–2.16)	0.5079
Hypertension	0.90(0.55–1.49)	0.6875
Cataract	1.85(0.99–3.46)	0.0557
Glaucoma	1.08(0.40–2.88)	0.8794
Renal diseases	1.59(0.75–3.34)	0.2251
Dyslipidemia	0.73(0.39–1.38)	0.3335
COPD	1.44(0.41–5.06)	0.5667
Heart failure	0.85(0.25–2.84)	0.7902
Asthma	1.80(0.63–5.13)	0.2706
Liver diseases	0.70(0.22–2.24)	0.5497
Pneumonia	2.03(0.69–5.96)	0.1994
Arrhythmia	1.06(0.37–3.01)	0.9146
**Cause-specific hazard regression for death**		
**Anti-VEGF group vs. control group**	4.70 (4.42–4.99)	< 0.0001
**Age group**		
< 50	Ref	
51–60	0.97(0.91–1.04)	0.4596
61–70	1.14(1.07–1.22)	0.0002
71–80	1.92(1.79–2.06)	< 0.0001
81 +	3.88(3.6–4.19)	< 0.0001
**Sex, n (%)**		
Male	1.12(1.08–1.16)	< 0.0001
Female	Ref	
**Initial clinical stage**		
0–1	Ref	
2	1.63(1.51–1.76)	< 0.0001
3	1.74(1.63–1.87)	< 0.0001
4	4.40(4.06–4.77)	< 0.0001
**Comorbidity**		
Diabetes mellitus	1.14(1.08–1.20)	< 0.0001
Hypertension	0.96(0.92–1.01)	0.0947
Cataract	0.88(0.81–0.96)	0.0025
Glaucoma	0.91(0.80–1.04)	0.1823
Renal diseases	1.43(1.33–1.54)	< 0.0001
Dyslipidemia	0.82(0.78–0.87)	< 0.0001
COPD	1.46(1.31–1.63)	< 0.0001
Heart failure	1.46(1.33–1.60)	< 0.0001
Asthma	0.94(0.84–1.06)	0.3258
Liver diseases	1.07(0.98–1.16)	0.1288
Pneumonia	1.65(1.50–1.83)	< 0.0001
Arrhythmia	1.11(1.01–1.22)	0.0309

**Table 4:** Adjusted hazard ratios (AHR) and 95% confidence interval (95% CI) for new-onset retinal vein occlusion during the follow-up period for the study cohort, number of patients (*n* = 43,418)

	**AHR (95%CI)**	***p*-value**
**Cause-specific hazard regression for RVO**		
**Anti-VEGF group vs. control group**	2.04 (0.75–5.52)	0.1619
**Age group**		
< 50	Ref	
51–60	1.89(0.61–5.89)	0.2717
61–70	2.41(0.79–7.33)	0.1218
71–80	4.39(1.46–13.18)	0.0083
81 +	4.87(1.47–16.18)	0.0098
**Sex, n (%)**		
Male	1.15(0.70–1.89)	0.5747
Female	Ref	
**Initial clinical stage**		
0–1	Ref	
2	0.97(0.49–1.95)	0.9343
3	0.95(0.52–1.73)	0.8542
4	1.13(0.40–3.13)	0.8204
**Comorbidity**		
Diabetes mellitus	1.36(0.74–2.51)	0.3232
Hypertension	0.85(0.50–1.47)	0.5694
Cataract	1.69(0.82–3.50)	0.1572
Glaucoma	1.67(0.57–4.88)	0.3492
Renal diseases	1.64(0.74–3.62)	0.2221
Dyslipidemia	0.62(0.30–1.27)	0.1877
COPD	1.60(0.46–5.54)	0.4562
Heart failure	1.09(0.33–3.64)	0.8897
Asthma	1.75(0.61–5.00)	0.2997
Liver diseases	0.52(0.13–2.15)	0.3695
Pneumonia	1.45(0.43–4.88)	0.5453
Arrhythmia	1.18(0.41–3.37)	0.7549
**Cause-specific hazard regression for death**		
**Anti-VEGF group vs. control group**	4.68 (4.41–4.97)	< 0.0001
**Age group**		
< 50	Ref	
51–60	0.97(0.91–1.04)	0.4558
61–70	1.14(1.07–1.22)	0.0001
71–80	1.92(1.79–2.06)	< 0.0001
81 +	3.89(3.60–4.19)	< 0.0001
**Sex, n (%)**		
Male	1.12(1.07–1.16)	< 0.0001
Female	Ref	
**Initial clinical stage**		
0–1	Ref	
2	1.63(1.51–1.77)	< 0.0001
3	1.75(1.63–1.87)	< 0.0001
4	4.41(4.07–4.78)	< 0.0001
**Comorbidity**		
Diabetes mellitus	1.15(1.09–1.21)	< 0.0001
Hypertension	0.96(0.92–1.01)	0.0957
Cataract	0.88(0.81–0.95)	0.0019
Glaucoma	0.92(0.80–1.05)	0.2151
Renal diseases	1.43(1.33–1.53)	< 0.0001
Dyslipidemia	0.82(0.77–0.87)	< 0.0001
COPD	1.46(1.31–1.63)	< 0.0001
Heart failure	1.45(1.32–1.59)	< 0.0001
Asthma	0.94(0.84–1.06)	0.3217
Liver diseases	1.07(0.98–1.16)	0.1456
Pneumonia	1.65(1.49–1.82)	< 0.0001
Arrhythmia	1.11(1.01–1.22)	0.0342

## Discussion

To our knowledge, this is the first retrospective cohort study to discuss the association between use of systemic anti-VEGFs and subsequent RVO. We enrolled 6285 mCRC patients in the anti-VEGF group and 37,250 CRC patients in the control group without anti-VEGF treatment. We found the risk of RVO was not higher in the anti-VEGF group, compared to the controls. And overall, the risk of RVO was related to greater age and previous history of RVO.

Angiogenesis, the important process leading to the formation of new blood vessels, is one of the hallmarks of cancer. VEGF is the key angiogenic factor, especially in CRC pathogenesis. Since the advent of molecular targeting therapies for mCRC, the prognosis for patients with CRC has changed dramatically. Of all the molecules identified as leading to blood vessel formation, VEGF-A appears to be the main molecular driver of tumor angiogenesis. VEGF induces the activation of proteins such as urokinase, tissue-type plasminogen activator, plasminogen activator inhibitor-1 and matrix metalloproteinases, and binds toVEGFR1 andVEGFR2, which are predominantly expressed on the cell surface of several anti-apoptotic factors that promote tumor growth and tumor metastasis. Indeed, VEGF-A is overexpressed in the majority of solid tumors and for this reason is the primary target for anti-angiogenic drugs [8]. Four agents are used in anti-angiogenesis therapy for mCRC at present. Bevacizumab (Avastin®) is a recombinant humanized monoclonal antibody that blocks angiogenesis by inhibiting VEGF-A. Aflibercept (Elyea®), a recombinant fusion protein, acts like a VEGF-trap, binding the circulating VEGFs, including VEGF-A, VEGF-B and placental growth factor (PlGF). Ramucirumab (Cyranza®) is a fully human monoclonal antibody that is directed against the vascular endothelial growth factor receptor 2 (VEGFR2). Regorafenib (Stivarga®) is an oral multi-kinase inhibitor which targets oncogenic, stromal (PDGFR-β, FGFR) and angiogenic (VEGFR1-3, TIE2) receptor tyrosine kinase. In Taiwan, the medications reimbursed during our study period were bevacizumab and regorafenib, and since May 2021, ramucirumab has also been reimbursed as a third medication for the same indication. Therefore, the medications we included in our study were bevacizumab and regorafenib (Supplementary Table 2).

As the first and second line of treatment for mCRC, bevacizumab has been shown to improve survival significantly in mCRC patients [9]. The Bevacizumab Expanded Access Trial (BEAT) evaluated the safety and efficacy profile of bevacizumab in the treatment of mCRC patients, and concluded that serious adverse events included bleeding (3%), gastrointestinal perforation (2%), arterial thromboembolism (1%), hypertension (5.3%), proteinuria (1%) and wound-healing complications (1%) [10]. The result from this observational population-based study was similar to that of another real-world study (Bevacizumab Regimens: Investigation of Treatment Effects and Safety [BRiTE study]) [11]. A further Japanese study targeting a Japanese population also produced a similar result [12]. In several previous studies, bevacizumab has been reported to induce arterial hypertension, arterial thromboembolic events and gastrointestinal bleeding [13–15]. As for regorafenib, hypertension has also been reported as a common adverse event [16, 17]. Previous studies have suggested that VEGFs could cause simultaneous endothelial nitric oxide (NO) production and an upregulation of endothelial NO synthase expression, further leading to vasodilatation. Anti-VEGF agents therefore inhibit the synthesis of NO and contribute to increased vascular tone, vasoconstriction and decreased sodium ion renal excretion, which leads to elevated arterial pressure [18]. The hypercoagulable status of mCRC patients, in addition to anti-VEGF treatment, may cause further microangiopathy, leading to vasoconstriction, hemostasis of retinal vessels and ultimately RVO.

The risk factors for RVO in oncological patients remain unclear. A case report of renal cell carcinoma and subsequent metastases treated with axitinib developed bilateral retinal vein occlusion and subsequent cerebrovascular accident [19]. This case had no history of hypertension, and the potential risk factor for RVO was the medication, which was, by nature, a VEGF inhibitor. Another endometrial cancer patient who suffered from CRVO was exposed to lenvatinib/pembrolizumab combination therapy [20]. And a patient with disseminated metastatic renal cell carcinoma who received long-term treatment with sorafenib also developed bilateral CRVO. Recently, a patient who first presented as CRVO and CRAO was subsequently diagnosed with advanced mantle cell lymphoma [21]. Another case was reported as a breast cancer patient with anemia, which might be a risk factor for CRVO [22].

All the above examples were just case reports, and no large population-based study has been conducted. In our study, 0.14 percent of 6285 patients receiving anti-VEGF treatment and 0.19 percent of 37,250 controls had adverse outcomes. The absolute risk reduction was 0.04 (95% CI: -0.06%—0.15%). The number needed to harm (NNH) was 1635, indicating that the risk of adverse outcomes associated with anti-VEGF treatment was low. Overall, our study provided a more comprehensive and statistically significant assessment of the potential risks associated with anti-VEGF treatment in oncological patients, thus confirming the overall safety of this treatment approach.

Our study showed that the RVO risk was not different between the anti-VEGF group and the control group. However, we did observe some risk factors. Older groups, more than 70 years old, were at greater risk of RVO than younger ones, while gender, clinical cancer stage and other previous comorbidities were not associated with risk of RVO. An older population might be at greater risk since aging contributes to a greater number of risk factors for cardiovascular diseases, and risk factors for RVO. When considering the development of RVO, vessel atherosclerotic changes, oxidative stress and inflammation caused by aging are plausible and logical factors. According to our result, the most important risk factor for RVO is a history of RVO (Table 3). Possible mechanisms for recurrent RVO vary, though most are related to systemic hypertension, diabetes or atherosclerosis. However, some cases may be related to a hyper-coagulation status of retinal vessels. Hernández, J. L. et al. [23] reported cases of antiphospholipid syndrome (APS) and RVO, and they suggest that RVO could represent an organ-specific manifestation of APS. The mCRC patients with RVO histories might have hypercoagulable vessel instabilities. After anti-VEGF treatment, the inhibition of VEGF, leading to thrombosis or vasoconstriction of retinal vessels, would lead to recurrent RVO.

### Strengths

This study is the first large cohort study to investigate associations between systemic anti-VEGFs, including bevacizumab and regorafenib, and ocular adverse events such as RVO. Previously there were only case reports, which could hardly disclose any associations [4]. The nationwide and population-based design of this study has good statistical power and risk appraisal precision, especially since we used the TCR, which contains definite diagnoses of colorectal cancers. The survival, medication and procedure records in the TCR are precise, which challenges a shortcoming of most database studies. Our patients with RVO were identified throughout the country, and had undergone a comprehensive assessment and diagnosis by ophthalmologists or retinal subspecialists from an array of different hospitals, including clinics, district hospitals, regional hospitals and medical centers. Furthermore, we used a cohort study design to explore the RVO incidence in colorectal cancer patients receiving anti-VEGF treatment, and in controls. Finally, our results were reliable because the anti-VEGF group had much higher mortality than the control group, which was reasonable and compatible with the reimbursement policy for anti-VEGF treatment for colorectal cancer patients in Taiwan.

### Limitations

There were some limitations to our study. We excluded those who had AMI and stroke histories, which included 12,848 patients from the original cohort. Patients with AMI or stroke history tended to have more risk factors in common with RVO, so we excluded them to avoid the possibility of interaction. We also excluded 18,197 patients without initial clinical staging data for colorectal cancer, since we intended to use clinical stage as one of the covariates. This number was almost 20% of our original population, and their exclusion may have reduced the statistical power of our study. While the initial cancer stage was likely to be more advanced in the anti-VEGF group than in the control group, we assumed all patients in the anti-VEGF group to be at stage 4, due to the reimbursement regulations. The survival curve (Fig. 3) resembling patients with metastatic colon cancer lends credence to this assumption [24]. However, the registry system could not provide us with data on when the patients developed recurrent disease or advanced to stage 4. Since initial clinical stage was one of the important factors related to our treatment group and controls, missing data may have introduced some bias in this study. The number of cases with RVO outcome was too small, leading to a lack of statistical power. Also, there was a large gap in mortality rate between the anti-VEGF group and the control group (Fig. 3). The anti-VEGF group had lower survival, meaning that the patients might expire before they could develop RVO. Thus, we used the mortality rate as a competing risk factor and conducted a subgroup analysis, which delivered a similar result to our primary analysis. We also used a cause-specific hazard model to recheck the HR, which yielded similar results. We furthermore tried excluding those with RVO histories and the result remained robust. Due to the small case numbers of our outcomes, we did not attempt to sub-group by different anti-VEGF drugs in the treatment cohort.

## Conclusion

In this study, we observed a non-significant trend towards an increased risk of RVO among mCRC patients treated with anti-VEGF therapy. Additionally, we identified older age and a prior history of RVO as potential risk factors for the development of RVO. Our analysis yielded an NNH of 1635, suggesting that anti-VEGF therapy remains a relatively safe treatment option for mCRC patients. However, it is important for physicians to closely monitor patients for any changes in vision while undergoing anti-VEGF therapy. Given the limited number of RVO cases observed in this study, further large-scale population-based post-marketing surveillance studies are needed to confirm these findings.

## Supplementary Information

**Additional file 1.**

## Data Availability

The data that support the findings of this study are available from the NHIRD, but restrictions apply to the availability of these data, which were used under license for the current study and therefore are not publicly available. Data are, however, available from the authors upon reasonable request and with permission from the NHIRD.

## Abbreviations

CRC: Colorectal cancer
mCRC: Metastatic colorectal cancer
OS: Overall survival
PFS: Progression-free survival
EGFR: Anti-epidermal growth factor receptor
CRVO: Central retinal vein occlusion
BRVO: Branched retinal vein occlusion
RVO: Retinal vein occlusion
NHID: National Health Insurance Database
TCR: Taiwan Cancer Registr
DM: Diabetes mellitus
HRs: Hazard ratios
NO: Nitric oxide
APS: Antiphospholipid syndrome
CRAO: Central retinal artery occlusion
