# Supplementary material for: Risk of retinal vein occlusion in colorectal cancer patients receiving anti-vascular endothelial growth factors – a population-based cohort study
Source: BMC Cancer. 2023 Jun 14;23:545. doi: 10.1186/s12885-023-11037-4 (PMC10265868; doi:10.1186/s12885-023-11037-4)
Supplement: Supplementary file 1 — Additional file 1. [file 12885_2023_11037_MOESM1_ESM.pdf]

Supplemental table 1. ICD codes.

**Diseases**	**ICD-9**	**ICD-10**
Diabetes mellitus	250.XX	E10, E11
Hypertension	401.XX-405.XX	I10
Dyslipidemia	272.XX	E78
Heart failure	428.XX, 402.01, 402.11, 402.91	I50
Asthma	493	J45
Chronic obstructive pulmonary disease	496.XX	J44
Pneumonia	480-486	J12-J18
Ischemic heart disease	412-414	I24-I25
Arrhythmia	427.XX	I45, I47, I48, I49
Liver diseases	456.XX, 571.XX, 572.XX	K70-K77
Renal diseases	250.4X, 403.XX, 404.XX, 585.XX-589.XX, 283.11, 581.XX-584.XX, 753.0X, 753.1X	N17, N18, N19
Cataract	366.xx	H25-H28
Glaucoma	365.xx	H40, H42
Retinal detachment	361.0x, 361.8x	H33
Myopia	367.1, 360.21	H52.1
Ischemic stroke	433.X, 434.X, 437.1X	I63.x, I69.3x
Hemorrhagic stroke	430.X, 431.X, 432.X	I60.x, I61.x, I69.0x, I69.1x
Myocardial infarction (MI)	410.X, 411.X	I21.01, I21.02, I21.09, I21.11, I21.19, I21.21,

Supplemental table 2. ATC codes.

**Anti-VEGF for colorectal cancer**	**ATC code**
Bevacizumab	L01XC07
Ramucirumab	L01XC21
Regorafenib	L01EX05
**Anti-VEGF for Eye**	**ATC code**
Aflibercept	S01LA05
Ranibizumab	S01LA04
